# Health Insurance Schemes and Their Influences on Healthcare Variation in Asian Countries: A Realist Review and Theory’s Testing in Thailand

**DOI:** 10.34172/ijhpm.2024.7930

**Published:** 2024-03-10

**Authors:** Woranan Witthayapipopsakul, Shaheda Viriyathorn, Salisa Rittimanomai, Jan van der Meulen, Viroj Tangcharoensathien, Ipek Gurol-Urganci, Anne Mills

**Affiliations:** ^1^Department of Health Services Research and Policy, Faculty of Public Health and Policy, London School of Hygiene & Tropical Medicine, London, UK.; ^2^International Health Policy Program, Ministry of Public Health, Nonthaburi, Thailand.; ^3^London School of Hygiene & Tropical Medicine, London, UK.

**Keywords:** Health Insurance, Healthcare Variation, Medical Practice, Asia, Thailand

## Abstract

**Background:** Various features in health insurance schemes may lead to variation in healthcare. Unwarranted variations raise concerns about suboptimal quality of care, differing treatments for similar needs, or unnecessary financial burdens on patients and health systems. This realist review aims to explore insurance features that may contribute to healthcare variation in Asian countries; and to understand influencing mechanisms and contexts.

**Methods:** We undertook a realist review. First, we developed an initial theory. Second, we conducted a systematic review of peer-reviewed literature in Scopus, MEDLINE, EMBASE, and Web of Science to produce a middle range theory for Asian countries. The Mixed Methods Appraisal Tool (MMAT) was used to appraise the methodological quality of included studies. Finally, we tested the theory in Thailand by interviewing nine experts, and further refined the theory.

**Results:** Our systematic search identified 14 empirical studies. We produced a middle range theory in a context-mechanism-outcome configuration (CMOc) which presented seven insurance features: benefit package, cost-sharing policies, beneficiaries, contracted providers, provider payment methods, budget size, and administration and management, that influenced variation through 20 interlinked demand- and supply-side mechanisms. The refined theory for Thailand added eight mechanisms and discarded six mechanisms irrelevant to the local context.

**Conclusion:** Our middle range and refined theories provide information about health insurance features associated with healthcare variation. We encourage policy-makers and researchers to test the CMOc in their specific contexts. Appropriately validated, it can help design interventions in health insurance schemes to prevent or mitigate the detrimental effects of unwarranted healthcare variation.

## Background

 Variations in healthcare are characterised by differences in healthcare resources, performance of health organisations, and populations’ access to and utilisation of health services, for example, in health professionals and bed density, admission rate, vaccination coverage, rate of elective surgery, and compliance to clinical guidelines.^1^ Some degree of variation always exists because people have different health needs depending on heterogeneity of patients, disease severity, or patient preference.^2^ Observing extent and determinants of healthcare variation helps policy makers identify unwarranted variation ie, variation which cannot be explained by patient differences, which raise concerns about suboptimal quality of care, differing treatments for similar needs, or unnecessary financial burdens on individuals and health systems.^1,3^

 There has been a growing volume of literature demonstrating healthcare variation. In the United States, variation in healthcare delivery among Medicare beneficiaries has been an important subject of health services research for many decades.^4^ After the pioneering study, the US Dartmouth Atlas project, similar national health atlas projects across different models of healthcare followed, highlighting healthcare practice variation and/or health outcomes by geographical area including in England,^5^ Scotland,^6^ New Zealand,^7^ Australia,^8^ the Netherlands,^9^ and Spain.^10^ Over eight hundred studies from Organisation for Economic Co-operation and Development (OECD) countries were published from 1990 presenting healthcare variation in various clinical conditions across geography, hospitals, or physicians.^11^ It is generally difficult for practice variation studies to explain clearly the sources of variation and distinguish warranted and unwarranted variation, leaving a significant proportion of variation unexplained.^12,13^ Much of the previous literature revealed that availability of physicians, their knowledge, practice styles and specialties, and hospital resource levels, were common unwarranted causes.^13-18^ Some others mentioned healthcare seeking behaviour, regulations, and health insurance coverage.^19,20^

 This review focuses on health insurance and its potential influences on healthcare utilisation variation. We define health insurance by the OECD’s taxonomy, that is, a health financing mechanism to distribute financial risk associated with an individual’s healthcare expenditure that involves pooling costs over time through pre-payment and over people by risk pooling.^21,22^ Health insurance can come in the form of public health insurance (eg, tax-based schemes and social security schemes) or private health insurance (eg, mandatory private insurance, employment-related insurance, voluntary community-rated private insurance, and voluntary risk-rated private insurance).^21-23^

 Most OECD countries where voluminous healthcare utilisation variation research has emerged are well-resourced and many of them use national health insurance to purchase care for everyone. A single or harmonized healthcare purchasing mechanism applied to all people affects medical practice similarly and is unlikely itself to cause variation across population groups. Many countries in Asia, especially those which are highly resource-limited, are working on attaining universal health coverage (UHC) by providing one or many insurance plans for their residents. Where there are multiple insurance schemes such as in China, India, and Thailand, regulations often vary and may lead to variation in per capita healthcare expenses,^24^ and health provision and outcomes among patients with similar clinical diagnoses.^25-36^ Previous literature has suggested that certain characteristics of payment systems employed by healthcare purchasers such as payment rates, payment schedule, performance requirements, influence the behaviour of healthcare providers.^1,37^ This realist review aims to answer two questions: (1) what are features in health insurance schemes that contribute to healthcare utilisation variation in Asian countries? (2) what are mechanisms and contexts which allow these features to influence healthcare variation? We then use Thailand, a country in Asia which has a multiple health insurance system and ongoing efforts to reduce health insurance fragmentation, to apply and refine the framework for the Thai contexts.

## Methods

 A realist review approach was used due to the nature of research questions that this review aimed to address. Unlike a Cochrane style systematic review which attempts to answer precise research questions for simpler interventions eg, clinical treatments, this approach is suitable for reviewing empirical studies on complex social interventions, ie, to analyse how and why they work (or do not work) in particular contexts.^38,39^ The realist approach believes that “no deterministic theories can always explain nor predict outcomes in every context.”^40^ However, it looks for semi-predictable reoccurring patterns in particular contexts ie, semi-regularities, and aims to produce a context-mechanism-outcome configuration (CMOc), which explains how an intervention’s mechanisms interact with specific contexts to produce outcomes.^41^ Interested scholars and policy practitioners can apply the CMOc in other similar contexts. Our study had two stages. The first stage was to produce a middle-range theory for Asian countries. In the second stage, we tested our middle-range theory in the Thai context in order to produce a refined theory that could be used in Thailand. Overall, this study was conducted in five steps.

###  Stage I

####  Initial Theory Development

 We developed a rough initial theory based on preliminary review of the literature about insurance scheme features, and team discussion. From the literature, we discussed insurance features and their potential mechanisms to induce variation in healthcare utilisation. We summarised these in the initial theory. This theory would be compared with the findings from the data extracted from the systematic search and expert interviews.

####  Systematic Search

 We conducted a systematic search in four electronic databases covering medicine, public health, and healthcare policy and management namely Scopus, MEDLINE, EMBASE, and Web of Science. We used the PICOS (Population, Intervention, Comparator, Outcome, and Study design) approach to help develop key domains of search terms and eligibility criteria. Possible search terms for each domain were listed and attempted in the four databases with appropriate truncation, quote marks, and filters (ie, countries, language, and published years) (See Supplementary file 1). Finally, the search terms “health insurance” AND “variation,” which were broad but precise enough to yield relevant results, were selected. We also filtered Asian countries, English language, and years of publication where allowed. Hand searching from the reference lists of the included articles was also conducted.

 To identify the health insurance characteristics that can influence healthcare variation among the insured, we included studies that compared at least two insurance schemes or a single scheme with a policy change and presented the variation of medical practice, service provision, access to or utilisation of health services. Only studies that focused on Asian countries were included (based on the list of countries and territories in the Asia geographical region of the Statistics Division of the United Nations^42^). This list excluded Oceanic countries like Australia and New Zealand. Studies with multiple countries where at least one country was from Asia were included. We restricted the search to only research or review articles published in the English language between 2010-2021, the period after the World Health Report 2010: Health Systems Financing: the Path to Universal Coverage^43^ was published. We excluded studies that demonstrated the variation only between insured and uninsured people and also studies comparing schemes with a harmonised benefit package and provider payment mechanism, such as in Japan, because any observed variation would potentially be influenced by factors other than health insurance.

**Figure 1:**
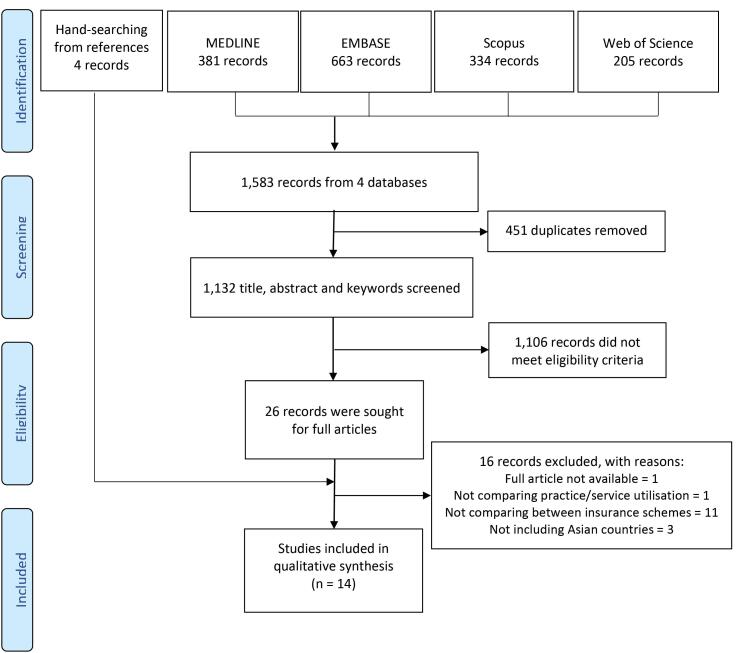
Screening Process Diagram.

 Search results from the four databases were exported to a reference manager software, Mendeley,^44^ where duplicates were removed. The remaining records were transferred to Rayyan (https://www.rayyan.ai/) which is a free web-based tool to facilitate systematic literature review conducted by multiple researchers. Two reviewers independently screened title, abstract, and keywords (where available) of every record and checked them against the eligibility criteria. Discrepancies were resolved by a third reviewer. Those records included by at least two reviewers were searched for full texts. Then the same selection process was repeated with full-text articles. The screening process was carried out by WW, SV, and SR. Figure 1 presents the number of articles at each screening stage.

####  Data Extraction and Synthesis of Systemic Literature Search

 We extracted information into an Excel spreadsheet on the included papers (authors, years of publication); study population and settings (countries, population groups, sources of data, and local settings); insurance schemes (schemes/plans); study objectives; services compared and key results relevant to variation in practice or utilisation. Then we imported all eligible full-text articles into NVivo software^45^ to analyse the contents in an inductive manner. We coded relevant texts to identify insurance features that contributed to healthcare variation including mechanisms that explain the observed variation and potential relationships between different features and mechanisms. This was an iterative process as we sought to capture semi-regularities that helped us to understand “what” contributed to practice variation, “how” it might, and “in what circumstance” it would. The findings were summarised in the CMOc format and served as a middle-range theory, which is “close enough to the data but abstract enough to apply to some similar situations.”^46^

####  Quality Appraisal of Included Literature

 We examined relevance and rigour to assess quality.^47^ Our inclusion and exclusion criteria served to ensure the relevance of evidence to the topic. During data extraction, we also checked whether the included studies provided information about insurance features, contexts and mechanisms useful for our theory building. We used the Mixed Methods Appraisal Tool (MMAT),^48^ which can accommodate various study designs, to appraise the methodological quality of included studies and assess rigour. Five quality criteria, focused on participant representativeness and risk of bias, were scored as yes, no, or cannot tell. Two researchers (WW and SV) studied the quality scoring together to ensure common understanding of the definitions, rating, and reporting manner before independently working on appraisal. The researchers compared their assessments and rechecked their answers for any disagreement. Remaining conflicts were resolved by discussion. We present MMAT appraisal results to provide information about credibility and trustworthiness but did not exclude any studies based on MMAT results as long as the study contributed to our theory building.

###  Stage II

####  Expert Interviews and Theory Refinement

 We conducted interviews of experts in Thailand to test our theory, ie, to consider whether the CMOc including the initial theory was applicable to the Thai context. Thailand has run multiple non-competing public health insurance schemes for many decades.^49^ There have been concerns about variation of practice and health outcomes, leading to ongoing attempts to merge or harmonise schemes.^50-52^ We purposively selected informants who have been involved in public insurance scheme harmonisation processes. The initial list of potential participants were members of public insurance scheme harmonisation subcommittee and working group. These groups were under the National Health Security Board and had mandates to carry out situation analysis and provide policy recommendations to promote a harmonised benefit package, provider payment methods, and management system across all public insurance schemes. We also used snowballing to identify additional participants. Table 1 presents participants’ profile. We started with the broad question about the sources of healthcare variation across public insurance schemes and used our initial theory and the CMOc to prompt when participants did not mention the features, mechanisms, or contexts that we found in the literature. The interviews were conducted in Thai via teleconference between August – October 2022. We coded the interview transcriptions to themes already listed in the middle-range theory’s CMOc and also allowed new themes to emerge. Quotations were translated to English by WW. Finally, we compared the initial theory, literature findings, and views of experts to produce a final refined theory for Thailand.

**Table 1:** Profiles of Thai Participants Contributing to the Refined Theory

**No.**	**Gender**	**Years of Experience**^a^	**Roles**	**Interview Date**	**Length (min)**
1	Female	2	Insurance scheme management; Harmonisation working group/subcommittee	4 Aug 2022	89
2	Male	18	Insurance scheme management; Harmonisation working group/subcommittee	11 Aug 2022	136
3	Female	25	Insurance scheme management; Harmonisation working group/subcommittee	27 Oct 2022	46
4	Female	32	Civil Society Organisation; Harmonisation working group/subcommittee	11 Aug 2022	32
5	Female	18	Insurance scheme management; Harmonisation working group/subcommittee	22 Aug 2022	74
6	Male	30	Civil Society Organisation; Harmonisation working group/subcommittee	23 Aug 2022	48
7	Male	30+	National public health reform committee; Harmonisation working group/subcommittee	6 Sep 2022	62
8	Male	12	Advisor to Ministry of Public Health	8 Sep 2022	28
9	Male	30+	Advisor to Ministry of Public Health	6 Sep 2022	37

^a^Relevant to insurance scheme harmonisation/variation.

 This paper follows the Realist And Meta-narrative Evidence Syntheses-Evolving Standards (RAMESES) reporting standards.^39^

## Results

###  The Initial Theory

**Figure 2:**
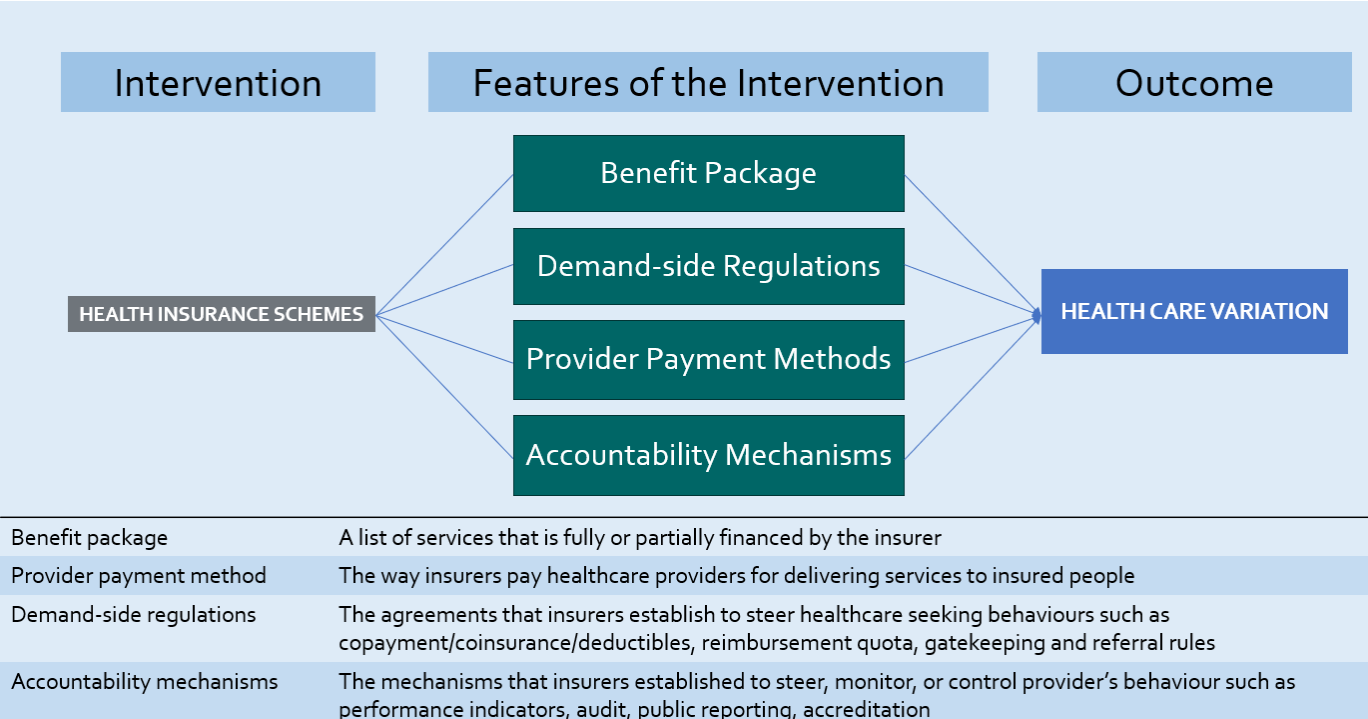
The Initial Theory Showing Key Features of Insurance Schemes That May Produce Medical Practice Variation.

 Our initial theory was based on a preliminary review of key papers on health insurance features.^37,53-57^ We identified four features in insurance schemes associated with healthcare variation (Figure 2). The most obvious feature is the benefit package, which is a list of services that are fully or partially financed by the insurer. Different insurance schemes have different benefit packages.^54^ Secondly, demand-side regulations such as cost-sharing policies, gatekeeping and referral rules applied by different schemes can differentiate the patient pathway and access to necessary and unnecessary care.^53,55^ When a service is not covered or some fees are required, people with a need for the service may face physical or financial access barriers, and therefore utilisation can be lower than normative need. Conversely, people may use services unnecessarily if they are covered although there may not be a normative need.^58^ Thirdly, provider payment methods significantly influence provider behaviours at institutional and individual health worker levels.^53,56^ In theory, proper payment designs can signal hospital managers and individual doctors to align behaviours with system-level goals, such as equity in access, quality of care, and efficiency, through the use of financial and non-financial incentives.^37,53^ For instance, with fee-for-service (FFS) payment, providers get paid for each service so there is no financial constraint on volume of services provided.^56^ On the other hand, capitation, diagnosis-related group (DRG), and global budget payments apply fixed budgets per person, case, or range of procedures over a period of time, so there is a financial constraint on volume of services provided.^53^ Finally, insurance agencies usually establish accountability mechanisms, such as medical audits, accreditation, quality and outcome framework, and mandatory reporting systems to monitor performance and behaviours of healthcare providers. Poor supervision systems may fail to signal when care provision is distorted from the optimal goal and lead to low quality care, under/overprovision, inequitable access, or fraud.^57^

###  Included Studies and Study Appraisal

 The search process found 1132 records from four databases after duplicates were removed. After screening the title and abstract, 1106 records were excluded. Screening full papers excluded another 16 papers, leaving 10 articles to be included in the study. We conducted handsearching from the reference list of these 10 articles and added 4 papers. In total, we found 14 papers that met the eligibility criteria.

 All included articles were observational studies comprising 11 cross-sectional, two cohort, and one case-control. A majority of articles provided good information in terms of representativeness of target population (13 out of 14 studies), appropriate measurement of exposure and outcome (14/14), accounting for confounders (13/14), and consistency of exposure status (14/14). A few studies acknowledged some methodology limitations such as potential biases arising from self-reported information, and unmeasured confounders. A common weakness across papers was that they often lacked information on data completeness (6/14). See Supplementary file 2 for MMAT rating of the 14 studies.

Table 2 summarises the study characteristics. Of the 14 studies, seven studies were from China, three from Indonesia, one each from South Korea and Thailand, and two were multi-country studies. Nine studies assessed overall outpatient and/or inpatient utilisation,^34,36,59-64^ while others focused on specific types of care namely preventive care,^65^ maternal care,^66^ Western and Chinese medicine,^67^ breast and cervical cancer screening,^68^ hip fracture hospitalisation,^69^ and tonsillectomy.^70^

**Table 2:** Characteristics of 14 Included Studies

**Authors (Year)**	**Countries**	**Local Contexts/Data Sources**	**Study Objective(s)**	**Services Compared**	**Insurance Schemes Compared**	Relevant Findings*	Insurance Features**
Li and Zhang (2013)^59^	China	Zhejiang (one of the richest provinces) and Gansu (one of the poorest and most rural provinces) The two provinces vary significantly in terms of geographic location, economy, and culture	To examine how different types of health insurance programs affect the following healthcare utilisation outcomes among the older people in China	OP and IP care utilisation	UEBMI URBMI NRCMS No health insurance	Compared to the uninsured, UEBMI members were 12.6% more likely to have OP visits in Zhejiang and 15.8% to be hospitalised in Gansu. NRCMS was 10.1% less likely to have OP visits in Gansu.	Beneficiaries; cost-sharing policies; benefit package; contracted providers; budget size; administration and management
Fang et al (2014)^65^	China	9 provinces (Heilongjiang, Liaoning, Jiangsu, Shandong, Henan, Hubei, Hunan, Guangxi, and Guizhou) in China that differ substantially in economic development, public resources, and healthcare indicators	To compare the three health insurance plans, examining whether they have created disparities in healthcare utilisation and expenditures	Preventive healthcare services Doctor visits	UEBMI URBMI NRCMS No health insurance	UEBMI members were 1.64 times more likely to receive preventive care than NRCMS members. No significant disparities in seeing a doctor when sick.	Beneficiaries; cost-sharing policies; benefit package
Chen et al (2018)^61^	China	Sichuan province which is a relatively undeveloped province in southwestern China	To compare the utilisation of OP and hospitalisation services and measure the inequity of the utilisation due to household income among elderly individuals enrolled in the three medical insurance schemes	OP and IP care utilisation	UEBMI URBMI NRCMS	OP utilisation was almost identical in all schemes. NRCMS had a lower hospitalisation rate than UEBMI and URBMI.	Benefit package; budget size
Fu et al (2018)^60^	China	450 communities/villages in 150 counties/districts	To assess the present inequality and horizontal inequity for health service use among the elderly in China To identify the main determinants associated with the disparity	OP and IP care utilisation	UEBMI URBMI NRCMS Other health insurance Two kinds of health insurance No health insurance	Overall, both probability and frequency of OP and IP utilisation showed pro-rich inequity. UEBMI contributed to pro-rich inequity in frequency of OP (17.58%) and IP (13.40%). NRCMS reduced pro-rich inequity for both OP (-35.80%) and IP care (-5.89%). URBMI showed a minimal contribution to inequity on both OP and IP.	Beneficiaries; cost-sharing policies; benefit package
Huang et al (2019)^64^	China	12 cities of Shandong province in China	To explore the seasonal and monthly patterns, weekly variations, and the holiday eﬀect on OP visits for type 2 diabetes mellitus patients, as well as the inﬂuence of gender, age, and insurance type on variations	OP visits among type 2 diabetes mellitus patients	UEBMI URBMI	Insurance type had a significant impact on OP visits. The effects varied across seasonal categories (seasons, monthly, weekly, holidays).	Beneficiaries
Fu et al (2020)^63^	China	28 provincial administrative units in China	To explore the changing trend in the unfairness of probability and frequency of IP services utilisation among middle-aged and elderly individuals with NCDs in China and analyse the main factors leading to this trend	IP services utilisation	UEBMI URBMI NRCMS Other health insurance No health insurance	UEBMI contributed to the decline in pro-rich inequity while NRCMS contributed to the decline in pro-poor inequity.	Beneficiaries; benefit package
Shi et al (2021)^36^	China	Pudong New Area in Shanghai	To explore the characteristics and factors inﬂuencing patient choices and the resulting utilisation of different levels of public medical institutions among elderly IPs with chronic diseases	Hospital admission at different levels of public medical institutions	UEBMI URBMI Out-of-pocket Others	URBMI were more likely to use community health centres than secondary and tertiary hospitals compared to UEBMI.	Cost-sharing policies; contracted providers
Sparrow et al (2017)^62^	Indonesia	262 out of 497 districts representing approximately 58% of the Indonesian population in 2010	To investigate the effect of local healthcare ﬁnancing schemes on access to healthcare and ﬁnancial protection	OP and IP care utilisation	District healthcare financing schemes (a great deal of variation in the design, such as coverage, benefit packages and provider contracting)	Choice of providers appeared an important source of impact heterogeneity.	Benefit package; contracted providers
Hartwig et al (2019)^66^	Indonesia	262 out of 497 districts representing approximately 58% of the Indonesian population in 2010	To investigate the eﬀectiveness of the local healthcare ﬁnancing schemes in improving access to maternal healthcare services	Maternal care	District healthcare financing schemes (a great deal of variation in the design, such as coverage, benefit packages and provider contracting)	Explicit benefit package had a positive effect. Contracting local rather than national healthcare providers increases the effects on maternal care.	Benefit package; contracted providers
Mulyanto et al (2019)^34^	Indonesia	497 districts within 33 provinces in Indonesia (whole country)	To investigate geographical inequalities in healthcare utilisation across 497 districts in Indonesia and whether compositional factors – wealth, education, health insurance – contribute to such inequalities	OP and IP care utilisation	Civil servant insurance Public insurance for the poor Private health insurance No health insurance	Unexplained district variation was substantial, comparable to that associated with health insurance.	Beneficiaries; benefit package
Kim and Roe (2019)^67^	South Korea	Pooled data representing the whole nation	To explore the possibility that Korea’s unique coverage structure of private health insurance causes conventional medicine to be more accessible than traditional medicine	Doctor visits of conventional medicine (Western) and traditional medicine (Chinese)	National health insurance *with* FFS-PHI National health insurance *without *FFS-PHI	Having FFS-PHI increased conventional doctor visits by 6.6%. No significant variation observed for traditional doctor visits.	Benefit package
Mukem et al (2015)^68^	Thailand	76 provinces covering municipal (urban) and non-municipal (rural) areas (whole country)	To understand the relationship between the use of breast and cervical cancer screening and socioeconomic and health insurance status To identify reasons for refraining from screening across Thai women in different groups	Breast and cervical cancer screening	UCS CSMBS SHI Private health insurance No health insurance	CSMBS had higher odds of having mammogram (1.50 in 2007; 1.75 in 2009) and cervical cancer screening (1.66 in 2007 and 1.56 in 2009) than UCS. SHI had higher odds of having mammogram than UCS (1.36 in 2009).	Beneficiaries
Kim et al (2021)^69^	Japan, South Korea, and Taiwan	Data from nation-wide databases	To examine the variations in LOS and related costs for IP hip fracture surgery care in Japan, Korea, and Taiwan	Hip fracture IP care	SHI systems in three economies	Treatment approaches varied across three systems. High supply capacity contributed to longer LOS in Japan and Korea. DRG payment in Taiwan contributed to shorter LOS.	Provider payment methods
Crowson et al (2017)^70^	31 countries	Data came from the 2015 OECD Health Statistics surgical procedures database	To analyse variation in tonsillectomy procedure rates between healthcare system types around the world	Tonsillectomy	Statist SHI National health insurance National health service Private health insurance SHI Social-based mixed type	SHI had higher rate than other types. Private care provision had higher rate than state provision.	Contracted providers; administration and management

Abbreviations: UEBMI, Urban Employee Basic Medical Insurance; URBMI, Urban Resident Basic Medical Insurance; NRCMS, New Rural Cooperative Medical Scheme; OP, Outpatient; IP, inpatient; NCDs, Non-communicable diseases; FFS-PHI, fee-for-service private health insurance; UCS, universal coverage scheme; CSMBS, Civil Servant Medical Benefit Scheme; LOS, length of stay; DRG, diagnosis-related group; OECD, Organization for Economic Co-operation and Development; SHI, social health insurance. * We reported only findings on health care utilisation. Other findings such as on expenditure were not reported. ** Insurance scheme features that were reported as potential sources of health care variation.

###  Findings

 We identified seven health insurance features which reportedly contributed to healthcare variation, and we categorised the mechanisms of variation as demand and supply side mechanisms, in line with previous research on healthcare variations.^1^ Scheme beneficiaries, benefit package, and cost-sharing policies were associated with variation mainly through the demand-side while contracted providers, provider payment methods, and budget size had influence mainly through the supply side. Administration and management involved both demand- and supply-side mechanisms influencing healthcare variation.

Table 2 also summarises the insurance scheme features of healthcare variation reported by the 14 included studies. The following paragraphs describe how studies related these insurance features to observed healthcare variation and mechanisms underlying them.

###  Beneficiaries

 Seven studies, from China, Indonesia, and Thailand, raised concerns about structural differences in characteristics of schemes’ beneficiaries such as occupation and area of residence (eg, urban/rural). These personal demographic and socioeconomic characteristics influenced health seeking behaviours, access, and utilisation.^65,68^ There were a number of mechanisms that influenced utilisation patterns.

 One mechanism was travel cost to health facilities borne by beneficiaries. In China, public health insurance schemes reflected where people lived. The New Rural Cooperative Medical Scheme (NRCMS) was for people who lived in rural areas while the Urban Employee Basic Medical Insurance (UEBMI) and the Urban Resident Basic Medical Insurance (URBMI) were for urban populations. Hence, travel costs from home to health facilities of the NRCMS beneficiaries were larger than for the enrolees of UEBMI and URBMI and reduced the former’s utilisation of services.^59^ In Indonesia where insurance schemes were run by local district governments, wide variations in travel expenses were observed due to varying distance to healthcare facilities in Indonesian districts, which could partly explain the disparities of service utilisation.^34^

 Occupation of the beneficiary had several mechanisms to increase variation. A Chinese study on diabetes patients explained that because the URBMI patients were mainly farmers, their doctor visits during intense farming periods were lower than those of the UEBMI counterparts.^64^ Two other Chinese studies suggested another mechanism involving occupation, namely doctor-driven supplier-induced demand in the UEBMI scheme because doctors knew that UEBMI members were employed and had regular income making cost-sharing more affordable.^60,63^ In Thailand, the universal coverage scheme (UCS) covered the population not covered by formal sector schemes for civil servants and private sector employees, so a large proportion were farmers who had lower education and income levels. Consequently, it was hypothesised that their lower level of health literacy led to sub-optimal uptake of free-of-charge preventive services.^68^ As in the Chinese study,^60^ it was argued that highly educated people had better awareness and communication skills, and good relationships with healthcare providers meant they knew their benefits and could make informed choices about care utilisation.

###  Benefit Package

 Nine papers considered that benefit package was a source of variation. Consistently across studies, the breadth of the benefit package affected access to services. The main mechanism appeared to be through financial risk shouldered by people protected by varying coverage. The number of services and the degree of cost coverage in the benefit package determined the size of financial barriers that scheme members would need to face. Individual affordability and physical access then affected the decision to seek and receive care. In China, UEBMI was significantly more generous compared to URBMI and NRCMS.^59-61,65^ The NRCMS’s benefit generally focused on expensive inpatient and outpatient care for many chronic conditions^65^; and while mainly targeting hospitalisation, it still had narrow inpatient benefits.^60^ This meant enrolees paid a large proportion of medical care costs out-of-pocket. The evidence suggested that such benefit package variation led to disparities in service received, favouring UEBMI members and disadvantaging especially the low-income population in NRCMS.^60,61,63,65^

 A similar picture was seen in Indonesia. The study indicated that, overall, covering basic services and medicines in the benefit package was positively associated with outpatient utilisation.^62^ However, the study also revealed that including advanced services in the context of a limited budget reduced overall outpatient utilisation.^62^ This was because advanced services consumed budget that could have been used for less costly basic services. The Indonesian experience also demonstrated that explicit inclusion of specific services did improve access, as in the examples of antenatal care and delivery assistance.^66^ In South Korea, having voluntary additional private insurance, which extended coverage for those not protected by the national health insurance plan and paid the cost sharing required by public insurance, increased doctor visits for Western medicine but did not affect Chinese medicine use. The study explained that this was possibly due to limited supply of traditional services.^67^

###  Cost-Sharing Policies 

 Four studies identified cost-sharing rules as a reason for variation. The Chinese experience provided substantial insights on cost-sharing policies. The UEMBI offered a more generous reimbursement rate compared to the other two schemes.^60^ Higher deductible and coinsurance rates, lower reimbursement ceiling, and retrospective reimbursement to beneficiaries appeared to be important access barriers among the members of NRCMS and URBMI.^59,65^ Different cost-sharing rates across level of health facilities additionally influenced where patients sought healthcare. Compared with those using the more generous UEBMI, inpatients using URMBI were more likely to go to community health facilities than secondary hospitals and tertiary hospitals. The authors presumed that the observed pattern was because the self-pay rates were higher in secondary hospitals and tertiary hospitals than in community hospitals.^36^

###  Contracted Providers

 Five studies discussed the issue of contracted healthcare providers. Insurance schemes usually indicated where their members could use healthcare services. Level, type, and location of providers affected the degree and pattern of service utilisation. In Indonesia, the heterogeneity of providers contracted by district health insurance schemes contributed to the schemes’ impacts on outpatient and inpatient care.^62^ From the study, the schemes which prioritised public or private providers located in districts were more effective in increasing utilisation than those which contracted provincial and national hospitals.^62^ Another study observed similarly that contracting providers of more advanced medical care at provincial or national level reduced access to maternal care.^66^ The study focused on 31 OECD countries found that health systems with private care provision had higher tonsillectomy rates than those with state-provided care.^70^ Most countries in the former group had national or social health insurance (SHI) systems while most in the latter had national health service systems ie, systems where financing, provision, and regulations were largely controlled by states. In China, the URMBI enrolees were more likely to use inpatient services at community health facilities than secondary hospitals and tertiary hospitals, compared with the UEBMI,^36^ possibly in part because patients experienced difficulties in reimbursement when they used health facilities in other counties or cities.^59^

###  Provider Payment Methods

 We found scarce information on provider payment mechanisms in our included studies and no study compared different payment methods in a single country. A study on variations in hip fracture-related inpatient care in Japan, Korea, and Taiwan discussed provider payment methods as a reason for practice variation.^69^ Japan and Taiwan used a DRG system while South Korea used FFS. The authors explained that DRG payment sent a strong incentive for hospitals to discharge patients early in Taiwan but not in Japan where quality improvement was given priority over cost-containment. In South Korea, FFS schemes incentivised hospitals to provide more services and keep patients longer.^69^

###  Budget Size

 Two studies in China posited that the available budget of the scheme affected overall service utilisation via its implications for individual contributions. In the Chinese insurance schemes, a significant source of funding came from individual contributions deducted at a specific percentage of personal income for UEMBI or collected at premium rates defined by local governments for URMBI and NRCMS.^61^ In poorer provinces where the income level was generally low, the budget for health was smaller than in richer provinces, and reimbursement rates lower. Gansu, one of the poorest provinces, had an outpatient reimbursement rate of only 17% while that of Zhejiang, a rich province, was 63%. A small budget might thus prevent some members from using necessary services when reimbursement rates were low.

###  Administration and Management

 Six studies discussed some mechanisms of administration and management. The first is level of decision. In decentralised policy contexts, such as in China and Indonesia, decisions on, for example, target population, benefit package, cost-sharing policy, provider payment, reimbursement quota, staff incentive, and other subsidies, were made at local level. Therefore, different regions, districts, or counties had their own scheme policies leading to substantial variations of utilisation rates and patterns across geographies.^34,59^ For instance, Papua was a special autonomous region in Indonesia where local government could decide on insurance coverage, transportation subsidies, supply-side strengthening policies, and additional staff incentives.^34^ The second mechanism is the nature of the entities involved in different management functions. The study in 31 OECD countries concluded that systems with societal regulation and financing (regulation and finances that were controlled by statutory bodies outside direct government control), as compared to state regulated or state financed systems, had a higher tonsillectomy procedure rate and the private health insurance system of the United States was among the highest rates.^70^

 Based on the evidence above, we summarised findings from the literature as CMOc, depicting seven insurance features and underlying mechanisms of variation. At this stage, the CMOc serves as a middle-range theory, which awaits further testing in the settings where the theory is intended to be used (Table 3, Source L).

**Table 3:** Summary of Identified Context-Mechanism-Outcome Configuration

**Insurance Scheme Features**	**Contexts**	**Mechanisms**	**Outcomes (Variations in Care)**	**Thai Experts’ Opinions**	**Source**
**Demand Side**					
**Beneficiaries ** Note: Insurance schemes which target specific population groups, eg, urban/rural areas, formal/informal sector, will result in specific patterns of age, education, occupation profiles.	Area of residence: beneficiaries residing in rural areas	Beneficiaries residing in rural areas have higher transport and indirect costs (eg, wage loss) for visits.	Lower healthcare utilisation (underuse)	Agree especially for advanced care services which are normally concentrated in cities	L
	Area of residence: beneficiaries residing in populous areas	Beneficiaries residing in populous areas face overcrowded hospitals which are less able to appropriately manage basic chronic care.	Lower healthcare utilisation (underuse)	Added by Thai experts	E
	Employment/occupation: beneficiaries in formal sector	Beneficiaries working in the formal sector are more likely to have higher and steady income levels. Clinicians knowing that the patient has a formal job/steady income could induce demand.	Higher healthcare utilisation (overuse)	Diverse opinions. Some agree, some disagree, some believe that it only happens when more care leads to more revenues	L
	Employment/occupation: beneficiaries in formal sector	Beneficiaries working in the formal sector are more likely to have higher education levels. Higher education levels are associated with increased health awareness and informed decision making and better communication skills & relationship with providers.	Optimal (or potentially higher) healthcare utilisation	Agree	L
	Employment/occupation: beneficiaries in agricultural sector	Farmers avoid seeking care in agricultural seasons eg, harvest period.	Seasonal variations in healthcare utilisation	Agree	L
	Age: beneficiaries are working age group	Beneficiaries of a scheme that has only working age members are healthier than schemes that include young children and elderly.	Lower healthcare utilisation	Added by Thai experts	E
Benefit package	Benefit package includes basic services and medicines	Beneficiaries can access these services with existing benefits package with no financial burden.	Higher healthcare utilisation for basic/ preventive care	Agree	L
	Narrow benefit package includes expensive outpatient and inpatient service only	Beneficiaries may face financial burden if they use basic services.	Lower utilisation of basic care especially among the lower income group	Not applicable because benefit packages are not narrow for any scheme	L
	Benefit package explicitly includes a targeted service (eg, maternity care in Indonesia and non-essential drugs in Thailand)	Beneficiaries are aware of and can access these services.	Higher healthcare utilisation for the targeted service	Agree	L
	Benefit package includes expensive/advanced care in resource limited settings	Expensive services consume budget that could have been used for basic services for more people.	Lower overall utilisation of care	No evidence that this happens	L
	Opportunity to buy extra insurance to extend coverage	People who opt for extra insurance can access service with no financial burden when the supply of service is adequate.	Higher healthcare utilisation for the services with adequate supplies	Agree	L
Cost sharing policies	High deductible and coinsurance rates, low reimbursement ceiling and retrospective reimbursement	Beneficiaries may not be able to afford services with high levels of cost-sharing or requiring advance payment.	Lower healthcare utilisation Effect modified by personal income/wealth with further reduction in utilisation for beneficiaries with lower income/wealth levels	Agree	L
	Different cost-sharing policies across levels of health facilities	Beneficiaries choose health facilities with lower levels of cost-sharing (eg, community health centres vs. secondary/tertiary hospitals).	Variations in healthcare utilisation across providers Higher healthcare utilisation in the community (where cost sharing is likely to be lower)	Not applicable because cost-sharing rates are the same across facility levels	L
Contracted providers	Schemes administered by local governments, contracting local providers (public or private)	Beneficiaries choose local health facilities where possible due to difficulties in reimbursement when they access care outside residential districts.	Lower healthcare utilisation in providers outside of the beneficiary’s residential area	Not applicable because patients are not required to pay upfront	L
Administration and management	Schemes administered by local governments	Decentralised decision-making on target population, benefit package, cost-sharing policy, reimbursement quota, and other subsidies.	Variations in healthcare utilisation across geographical areas	Diverse opinions. Some agree, some argue that local governments’ role is very limited	L
	Schemes prone to political interference	Some schemes may be more prone to political interference than others, especially those targeting vulnerable groups or the majority. Some demand-side features such as patient pathway, cost-sharing, benefit package can be manipulated in political campaigns.	Variations in healthcare provision across insurance schemes	Added by Thai experts	E
**Supply Sde**					
Contracted providers	Contracting local providers (public or private)	Scheme reduces physical access barriers to care.	Higher primary care utilisation	Agree	L
	Contracting national and provincial providers	Providers are specialised in advanced care.	Lower maternal care utilisation	Agree	L
	Contracting referral/university hospitals	Providers tend to provide more intensity of care when they are paid by care volume.	Higher healthcare utilisation for members of insurance schemes which pay by care volume	Added by Thai experts	E
	Hospitals minimising financial risk	Hospital managers encourage physicians to provide more services when hospitals are paid by care volume, and restrict expensive care options when paid by case or person.	Variations in healthcare utilisation across insurance policies	Added by Thai experts	E
Provider payment methods	Using case-based payments (eg, DRGs) or capitation	Providers need to be efficient in resource use given limited payment per case or per person.	Lower amount/intensity of services per case or per person	Agree	L
	Using FFS	Providers get more payment when they provide more services.	Higher amount/intensity of services per case	Agree	L
	Reimbursement rate	Providers make more profit when the reimbursement rate is higher.	Higher amount/intensity of services for members of the insurance offering generous payment rates	Added by Thai experts	E
Budget size	Small insurance budget	Schemes with limited budget are unable to cover all needed services although they are in benefit package. Some people are forced to pay the full cost when the budget is finished.	Lower healthcare utilisation	No evidence that it happens	L
Administration and management	Schemes administered by local governments	Decentralised decision-making on supply-side policies and staff incentives.	Variations in healthcare provision across geographical areas	Agree but limited role of local governments	L
	State regulation and financing system	State regulation is more effective in controlling provider behaviour than societal agreement by non-governmental organisations. Providers are less likely to provide more than necessary services.	Lower healthcare utilisation	Not applicable because regulation and financing system of public insurance schemes in Thailand is managed by governmental organisations	L
	Schemes with different composition and source of management board members	Composition (representatives from different parties) and selection (ie, election, appointment) affect high-level decisions on goals and objectives of schemes.	Variations in healthcare provision across schemes	Added by Thai experts	E
	Schemes with accountability mechanisms	Accountability mechanisms such as audit, performance indicators, public reporting can steer provider’s behaviour towards expected outcomes.	Variations in healthcare provision across schemes	Added by Thai experts	E

Abbreviations: FFS, fee-for-service; DRG, diagnosis-related group Light grey shade indicates a refined theory for Thailand; Dark grey shade indicates mechanisms not applicable to Thailand; L indicates mechanisms identified by literature; E indicates mechanisms identified by Thai experts.

###  Findings From Expert Interviews

 We explored the views of nine Thai experts on sources of healthcare variation across public insurance schemes in Thailand to test our theory in the Thai context.

 Overall, the experts concurred with most findings from the literature, had mixed opinions with a few, and found some mechanisms not applicable to the Thai context (Table 2). The majority highlighted that differences in payment methods and reimbursement rates were key reasons for variation. In Thailand, the civil servant scheme paid outpatient service by FFS while the other larger schemes mainly used capitation with some high-cost items reimbursed by fixed fees. The civil servant scheme also offered higher reimbursement rates for many medical procedures and devices.

 “*Not only the payment method, but reimbursement rate also affects practice. For example, artificial lens or dental implants, there are many options. Even though the payment method is the same but if the reimbursement rates are different, the quality will be different” *(Expert No. 8)

 For benefit package, they generally agreed that a more comprehensive package increased total utilisation, but they did not see any evidence that including expensive services would consume the budget and lead to lower utilisation as a whole. The informants also agreed that when a service was explicitly covered, it did improve access because both doctors and beneficiaries were aware that they could provide or use the service. In Thailand, services were often made explicit when they were paid in addition to capitation or case-based payment, hence, this also offered extra payment to providers.

 “*There is nothing that we do not cover in capitation and DRG payment. We have some 200 medical devices that we pay on top, which means unbundling from DRG…Some doctors misunderstand that items not in reimbursement price list are not covered (by insurance). So awareness is also a barrier” *(Expert No. 5).

 Regarding providers, the experts agreed that contracting both public and private providers improved access to care because it shortened waiting time in contrast to relying only on overcrowded public hospitals. However, one expert pointed out that the civil servant group, despite being restricted to public facilities for most services, had higher utilisation rates than other schemes’ beneficiaries because the civil servant scheme’s payment rates were higher. Provider levels also affected utilisation patterns of patients differently by scheme. An expert explained that a health centre or a community hospital would treat people similarly given limited care options. However, a referral hospital or university hospital might provide more intensity of care (such as computed tomography scan) for civil servants because they had a wider range of services and knew that the scheme would pay for all procedures.

 “*Having more private providers improve access especially for elective surgeries or prevention services and antenatal care...” *(Expert No. 1).

 “*There are other factors. Although the civil servants are restricted to only public hospitals, the higher payment rate may make their access greater than people from other schemes” *(Expert No. 5).

 “*If it is a community hospital, I don’t think there are differences between insurance schemes because they don’t have that many varieties, for example, medicine options” *(Expert No. 4).

 Thai experts had diverse opinions about supplier induced demand in the context of stable patient income. Some believed that doctors would not know or consider patients’ economic status when they treated them.

 “*I don’t think so. Doctors wouldn’t know how much their patients earn. They would just look at the patient’s insurance rather than how rich their patients look” *(Expert No. 1).

 Others thought that this phenomenon might affect the civil servant group only where their scheme paid fee for service, and not members of other schemes which paid capitation or DRG.

 Some CMOc found in the literature did not seem to exist or be applicable to the Thai context. Thailand used the same patient cost-sharing rate across facility types; and, except for the civil servant group, required people to register and use care at specific facility networks. So members did not choose facilities based on cost-sharing rates. Another point was that insurance schemes were not managed by district governments: the budgets for schemes were set nationally and allocated directly to health facilities. Hence, there was no issue about patients accessing care outside their districts and local budgets had no influence on local access to care. Although local governments could make some decisions about extra benefits or additional staff incentives, these were very small.

 “*Our system is different. Decisions are made centrally. No matter where the hospital is, the same payment systems apply. Local governments do not have roles, for example, they do not own health facilities except in Bangkok” *(Expert No. 6).

 A few new issues were also raised. The first was hospital policy at the meso level which could influence individual physicians. Experts suggested that hospital managers might encourage physicians to provide more for the civil servants while restricting the use of expensive medicines among the beneficiaries of less generous schemes, to reduce the financial risk of hospitals. Second, the age structure of the beneficiaries affected healthcare seeking patterns. Thailand’s SHI scheme has only working-age members, so they tend to be healthier and consume care less than the other schemes’ members on average. This also influenced the provider’s capacity, as hospitals where a majority of clients were SHI beneficiaries adjusted their capacity to match clients’ demand, resulting in flexible operating hours but lower capacity to provide preventive care for young children and the elderly.

 “*We’re often told that doctors want to use this option [more expensive medical devices, drugs, or diagnostic tools] but the hospital managers do not allow because it will make a loss to the hospital. So do not use this and that” *(Expert No. 5).

 “*SHI members are working age. They fall ill less often than older people in Universal Coverage Scheme” *(Expert No. 6).

 “*The problem of SHI beneficiaries is they don’t want to take leave as it may affect their income. The scheme tried to help by requesting its contracted hospitals to open in the evening hours” *(Expert No. 6).

 The third issue concerns area of residence linked to the management issue. In Thailand, primary care and secondary care facilities are fully geographically covered at district level. Therefore, people residing in rural areas would face physical barriers or transport cost only when they need advanced care at provincial or regional hospitals. In contrast, people living in cities faced more limited access to basic chronic care (eg, diabetes management) than their rural counterparts because provincial hospitals were overcrowded, and additional facilities not made available to them. The primary care situation was worse in Bangkok where provider networks and referral systems were poorly managed despite very high density of public and private health facilities. This has been a challenge for SHI beneficiaries who tend to reside in populous areas where businesses are located.

 “*…Cities usually have more resources so urban residents should have greater opportunities. We have more doctors in Bangkok than in other cities but the management is so poor. … the management in Bangkok is a failure despite excessive resource. …Management is really important. Having more resources doesn’t guarantee that urban residents have better access to care” *(Expert No. 2).

 The last point is about scheme administration and management. Governing board composition and structure including number of members, selection, terms of service, and incentives were important factors that shaped the goals and objectives of the schemes (eg, whether or not there was strategic purchasing). According to the experts, degree of multi-stakeholder participation (representatives from different parties) and method of selection of representatives (eg, election, appointment) determined the level of responsiveness to beneficiaries. Political interference in a scheme’s policies was another important factor. The UCS which covered the majority of the population including the lower income group was often a target for political campaigns in health. These campaigns often reduced demand-side barriers or offered greater convenience such as accessing acute primary care anywhere, no gatekeeper for cancer services, one-time referral per illness episode, and changing registered hospital with immediate effect.

 “*Stakeholder participation is an important part of good governance. Universal Coverage Scheme identifies several stakeholder groups and uses a democratic method to select their representatives. The other two schemes do not have a clear participatory process, especially for the civil servant, it’s rather just picking up. The social health insurance has representatives from both employers and employees but it is more of a political competition among the candidates. Representatives determine the quality of their executive boards” *(Expert No. 7).

 “*The other factor is politics. We have to admit that it can interfere and change anything.…whenever the politicians become interested in health systems and try to engage with health policies, it affects both providers and patients including purchasers” *(Expert No. 2).

 Lastly, a few experts believed that accountability mechanisms such as medical and financial audit, public reporting, performance indicators, and quality accreditation could affect provider behaviour. Insurance schemes in Thailand applied these mechanisms to varying degrees. However, other experts argued that these mechanisms had only a slight impact on provider behaviour because most of them happened retrospectively ie, after services had been provided.

 “*The current system takes around 30 days to audit. By that time, the service has already been provided. It is not a real-time system. Quality outcome framework even takes longer time. These mechanisms do not have direct effects. It may be useful for the next years” *(Expert No. 5).

###  Theory Refinement for Thailand

 We refined the theory for Thailand by adding expert-identified mechanisms to the middle-range theory and removing those not applicable. In Table 3, the refined theory for Thailand consists of all statements shaded in green, in total 22 mechanisms.

## Discussion

###  Key Findings

 Our realist synthesis found seven features of health insurance schemes that can contribute to variation in healthcare. These features are beneficiaries, benefit package, cost-sharing policies, contracted providers, provider payment mechanisms, budget size, and administration and management. Many features influence one another. Administration and management of health insurance schemes affect all other features, and so influence healthcare practice through both demand- and supply-side mechanisms. The literature review suggested that the first three features influence variation mainly through demand-side mechanisms, notably patient characteristics such as age, sex, comorbidities, and their socioeconomic profiles (wealth, occupation, and education) which strongly link to their health literacy, healthcare seeking behaviours, and ability to pay. The other three features, namely contracted providers, provider payment mechanisms, and budget size influence variation primarily through supply-side mechanisms, including provider distribution, type of providers, and provider responses to financial (dis)incentives. However, a single mechanism can also have a complex interaction between demand and supply sides. For instance, an explicitly covered service in the benefit package increases awareness of both patients and doctors and stimulates use from both demand and supply sides. Additionally, either naming services or arranging additional payment for specific services in the benefit package incentivises providers to make them available, in effect reducing physical barriers for patient although this mechanism is not very clear from the study findings.

 Our middle range theory contains 20 mechanisms identified from 14 empirical studies conducted in both high-income and lower- and middle-income Asian settings. This middle range theory, together with inputs from the initial theory, requires testing in specific contexts and further refinement before policy use. We tested it in Thailand and developed the refined theory which discarded six mechanisms and added eight new mechanisms. The key differences between Thailand’s experience and literature findings stem from the Thai governance structure of health insurance schemes which are relatively more centralised than the contexts found in the literature. Therefore, issues related to local, decentralised management do not apply to Thailand, and some insights relating to meso- and macro-levels are added. We encourage policy-makers and researchers to test our middle-range theory (or Thailand’s refined theory) in the contexts where it is intended to be used.

###  Strengths and Limitations 

 To our knowledge, this review is the first realist review to explore sources of healthcare variation associated with multiple insurance schemes in resource-limited settings. We aimed to generate a theory to support further research or inform policy. However, our study is not without limitations. The major weakness is that we found an overall thin body of knowledge on our topic. Although we aimed to explore Asian contexts, our systematic search yielded 14 studies only from East and Southeast Asian countries, with China accounting for half of study settings, resulting in limited contextual information to compare effects, and possibly excessive influence of the Chinese system. Secondly, our findings lack comprehensive explanation about how an insurance feature influences variation. This may partly be due to our inclusion criteria which excluded discussion papers, opinion pieces, commentaries, case studies, theoretical papers or other types of grey literature which might offer insightful qualitative analyses of the mechanisms. Also, most papers focused on user perspectives, ie, access and utilisation, rather than provider perspectives, ie, medical practice. As a result, evidence on supply-side mechanisms is very limited. Even with a broad search strategy, scarce evidence was found and prevented us from formulating a comprehensive CMOc of healthcare variation, as intended. Thirdly, we mainly focused on variation between insurance schemes although variations within a single scheme also coexist and may confound the effects between schemes. Our research design was not suitable to differentiate such effects. Fourthly, our study used a qualitative approach, and thus cannot determine nor confirm causal relationships or associations between health insurance and observed variation. Finally, our findings help identifying sources of variation across insurance schemes, but do not consider the optimal level of service provision across all schemes.

###  Comparison With Existing Literature

 Previous research exploring sources of healthcare variation has not focused specifically on the attributes of health insurance schemes but rather has explored any factors across, most commonly, geographic areas.^13^ Much of the existing literature has highlighted the importance of discriminating between warranted (such as case severity, disease prevalence) and unwarranted variation (ie, underuse, overuse, and inappropriate use) but also has acknowledged the difficulties of doing so.^4,13,71-74^ Many sources of variation found by other researchers are similar to the mechanisms identified from our study, such as patient demographic and socioeconomic factors.^13^ Regarding supplier-induced demand, in the US, Wennberg, while agreeing that some services were induced by doctors, hospitals and clinics, or medical equipment availability, also argued that for services like elective surgery, physician preferences underlay much of decision-making.^2^ Previous studies identified physician practice style as an important influence on practice variation^1,73-76^ but our study found very little evidence about practice style in the context of variations associated with health insurance schemes. Moreover, policy-related factors such as preference for more cost-effective interventions, and environmental factors such as overcrowding hospitals,^74^ were not identified in our study. Likewise, research and policy papers discussing fragmentation of health insurance schemes are enormous but tended to focus on the differences of the features rather than healthcare utilisation variation. The commonly compared features are similar to those identified by our study, namely beneficiaries, benefit package, user charges, provider payment mechanisms, purchasing organisations, and also other features such as source of financing, contribution rates.^77-81^ In the literature, disparities in accessing health services due to multiple insurance schemes was often highlighted, but empirical evidence was limited and rarely linked to specific insurance features.

###  Implication for Policy 

 Our findings provide some evidence that multiple health insurance schemes are associated with practice variation. This variation may be warranted, but often is not and creates both inequities and inefficiencies. Multiple insurance schemes might not be easily avoidable as most countries have schemes targeting different populations such as the poor, formal and informal sectors.^82^ In such circumstances, harmonisation of benefit packages, provider payment methods and funding levels should be the way forward. Harmonisation facilitates a seamless transition of membership from one scheme to another scheme in a UHC era. Previous and ongoing health reforms merging or reducing the number of health insurance schemes have been evident in many countries around the world, for instance, China, Estonia, Indonesia, Peru, Poland, South Korea, Thailand, and Turkey.^79,82-85^ For countries at the initial phase of UHC development, a unified design of scheme features is an entry point to mitigate future healthcare variations even where there may be different schemes managed by different agencies. Oversight mechanisms, monitoring to capture unwarranted practice variation, and policy measures to prevent or minimise it, are recommended.

## Conclusion

 This realist review identified seven features of health insurance schemes that contribute to healthcare variation: beneficiaries, benefit package, cost-sharing policies, contracted providers, provider payment mechanisms, budget size, and administration and management. We captured various semi-regularities of the complex relationship between these features, their demand- and supply-side mechanisms, and contexts leading to healthcare variation in Asian settings. The theory testing in Thailand encountered both similarities and some differences, highlighting the importance of contexts. We recommend interested policy-makers and researchers to test our theory in other relevant contexts. The tested theory can signal areas of focus for policy interventions to reduce unwarranted healthcare variation in multiple health insurance systems. Future research may look at research settings outside Asia and compare the findings with this study. An epidemiological approach to test the association between health insurance schemes and healthcare variation, while differentiating the effects from within-scheme variation, is recommended.

## Acknowledgements

 We thank Josephine Borghi for the inspiration and useful advice about realist reviews, and Waritta Wangbanjongkun for coordinating the expert interviews in Thailand.

## Ethical issues

 Ethical approval for expert interviews was sought from the Institute for Development of Human Research Protection, Thailand (Certificate No. IHRP2021179 dated December 27, 2021) and London School of Hygiene and Tropical Medicine (Ethics Ref 27820 dated July 19, 2022).

## Competing interests

 Authors declare that they have no competing interests.

## Funding

 This review was funded by Health Policy and System Research Fellowship of the International Health Policy Program Foundation Thailand.

## Supplementary files

Supplementary file 1 contains search strategy and eligibility criteria.

Supplementary file 2 shows MMAT rating of the 14 studies.
