# Supplementary material for: Health Insurance Schemes and Their Influences on Healthcare Variation in Asian Countries: A Realist Review and Theory’s Testing in Thailand
Source: Int J Health Policy Manag. 2024 Mar 10;13:7930. doi: 10.34172/ijhpm.2024.7930 (PMC11608294; doi:10.34172/ijhpm.2024.7930)
Supplement: Supplementary file 1 — contains search strategy and eligibility criteria. [file ijhpm-13-7930-s001.pdf]

Article title: Health Insurance Schemes and Their Influences on Healthcare Variation in Asian Countries: A Realist Review and Theory's Testing in Thailand

Journal name: International Journal of Health Policy and Management (IJHPM)

Authors' information: Woranan Witthayapipopsakul[1,2]*, Shaheda Viriyathorn[2], Salisa Rittimanomai[2], Jan van der Meulen[1], Viroj Tangcharoensathien[2], Ipek Gurol-Urganci[1], Anne Mills[3]

[1]Department of Health Services Research and Policy, Faculty of Public Health and Policy, London School of Hygiene & Tropical Medicine, London, UK.

[2]International Health Policy Program, Ministry of Public Health, Nonthaburi, Thailand.

[3]London School of Hygiene & Tropical Medicine, London, UK.

***Correspondence to:** Woranan Witthayapipopsakul; Email: Woranan.witthayapipopsakul@lshtm.ac.uk

Citation: Witthayapipopsakul W, Viriyathorn S, Rittimanomai S, et al. Health insurance schemes and their influences on healthcare variation in Asian countries: a realist review and theory's testing in Thailand. Int J Health Policy Manag. 2024;13:7930. doi:10.34172/ijhpm.2024.7930

Supplementary file 1

Search strategy

The PICOS (Population, Intervention, Comparator, Outcome, Study design) approach was used to help developing key domains of search terms and eligibility criteria. We also filtered Asian countries, English language, and years of publication where allowed. The following terms were attempted.

**Domains**	**Qualification**	**Possible terms**
Population	Asian country populations†	Use filter where possible
Intervention	Different health insurance schemes<br>Any differences in insurance policies	insurance, health insurance, scheme*, program*, plan, system
Comparator	Population groups covered by different insurance schemes OR<br>Population groups targeted by different insurance policies	insurance, health insurance, scheme*, program*, plan, system
Outcome	Variation in medical practice, service provision, utilization rate	variation*, difference*, change*
Study design	Original research, Reviews such as systematic review, scoping review, rapid review	Use filter where possible

† Referred to countries and territories in Asia used by the Statistics Division of the United Nations (https://unstats.un.org/unsd/methodology/m49/)

Finally, the search terms 'health insurance' AND 'variation' were selected as they were broad but precise enough to yield relevant results. The exact terms and filters used in each database were reported below.

DATABASE	MEDLINE	EMBASE	SCOPUS	Web of Science
**KEYWORDS**				
**1**	variation	variation	variation	variation
**2**	health insurance	health insurance	health insurance	"health insurance"
**BOOLEAN**	AND	AND	AND	AND
**SCOPE**		title, abstract, keywords	title, abstract, keywords	topic
**FILTERS**				
**1**	2010-2021	2010-2021	2010-2021	2010-2021
**2**		English	English	English
**3**		Article	Article	Article
**4**		Human	Medicine	Asia (UN)
**5**			All open access	Open access
**6**			Asia (UN)	
**No of Hits**	381	663	334	205
**Date**	04-Oct-21	05-Oct-21	25-Oct-21	25-Oct-21

Eligibility criteria

Inclusion	Exclusion
1. studies that compared at least two insurance schemes or a single scheme with a policy change and presented the variation of medical practice, service provision, access to or utilisation of health services.<br>AND<br>2. studies that focused on Asian countries were included (based on the list of countries and territories in the Asia geographical region of the Statistics Division of the United Nations<br>AND<br>3. only research or review articles<br>AND<br>4. studies published in the English language between 2010-2021	1. studies that demonstrated the variation only between insured and uninsured people<br>OR<br>2. studies comparing schemes with a harmonised benefit package and provider payment mechanism, such as in Japan
