# Supplementary material for: Health Insurance Schemes and Their Influences on Healthcare Variation in Asian Countries: A Realist Review and Theory’s Testing in Thailand
Source: Int J Health Policy Manag. 2024 Mar 10;13:7930. doi: 10.34172/ijhpm.2024.7930 (PMC11608294; doi:10.34172/ijhpm.2024.7930)
Supplement: Supplementary file 2 — shows MMAT rating of the 14 studies. [file ijhpm-13-7930-s002.pdf]

Article title: Health Insurance Schemes and Their Influences on Healthcare Variation in Asian Countries: A Realist Review and Theory's Testing in Thailand

Journal name: International Journal of Health Policy and Management (IJHPM)

Authors' information: Woranan Witthayapipopsakul[1,2]*, Shaheda Viriyathorn[2], Salisa Rittimanomai[2], Jan van der Meulen[1], Viroj Tangcharoensathien[2], Ipek Gurol-Urganci[1], Anne Mills[3]

[1]Department of Health Services Research and Policy, Faculty of Public Health and Policy, London School of Hygiene & Tropical Medicine, London, UK.

[2]International Health Policy Program, Ministry of Public Health, Nonthaburi, Thailand.

[3]London School of Hygiene & Tropical Medicine, London, UK.

***Correspondence to:** Woranan Witthayapipopsakul; Email: Woranan.witthayapipopsakul@lshtm.ac.uk

Citation: Witthayapipopsakul W, Viriyathorn S, Rittimanomai S, et al. Health insurance schemes and their influences on healthcare variation in Asian countries: a realist review and theory's testing in Thailand. Int J Health Policy Manag. 2024;13:7930. doi:10.34172/ijhpm.2024.7930

Supplementary file 2

MMAT Screening Questions

S1. Are there clear research questions?

S2. Do the collected data allow to address the research questions?

Study No.	WW		SV	
	S1	S2	S1	S2
1	Yes	Yes	Yes	Yes
2	Yes	Yes	Yes	Yes
3	Yes	Yes	Yes	Yes
4	Yes	Yes	Yes	Yes
5	Yes	Yes	Yes	Yes
6	Yes	Yes	Yes	Yes
7	Yes	Yes	Yes	Yes
8	Yes	Yes	Yes	Yes
9	Yes	Yes	Yes	Yes
10	Yes	Yes	Yes	Yes
11	Yes	Yes	Yes	Yes
12	Yes	Yes	Yes	Yes
13	Yes	Yes	Yes	Yes
14	Yes	Yes	Yes	Yes

MMAT study design

Study No.	WW	SV	Question Set
1	Cross-sectional analytic study	Cross-sectional analytic study	3

2	Cross-sectional analytic study	Cross-sectional analytic study	3
3	Cross-sectional analytic study	Cross-sectional analytic study	3
4	Cross-sectional analytic study	Cross-sectional analytic study	3
5	Cohort study	Cohort study	3
6	Cross-sectional analytic study	Cross-sectional analytic study	3
7	Case-control study/Cross-sectional analytic study	Case-control study	3
8	Cross-sectional analytic study	Cross-sectional analytic study	3
9	Cross-sectional analytic study	Cross-sectional analytic study	3
10	Cross-sectional analytic study	Cross-sectional analytic study	3
11	Cohort study	Cohort study	3
12	Cross-sectional analytic study	Cross-sectional analytic study	3
13	Cross-sectional analytic study	Cross-sectional analytic study	3
14	Cross-sectional analytic study	Cross-sectional analytic study	3

MMAT Quality appraisal

3. Quantitative nonrandomized

Q1. Are the participants representative of the target population?

Q2. Are measurements appropriate regarding both the outcome and intervention (or exposure)?

Q3. Are there complete outcome data?

Q4. Are the confounders accounted for in the design and analysis?

Q5. During the study period, is the intervention administered (or exposure occurred) as intended?

Final result of quality rating after discussion

Study No.	Q1	Q2	Q3	Q4	Q5
1	Yes	Yes	Can't tell - There was no information of completeness of outcome data.	Yes	Yes
2	Yes	Yes	Yes	Yes	Yes
3	Yes	Yes	Yes	Yes	Yes
4	Yes	Yes	Yes - The authors reported some missing data (213/8049) which was acceptable.	Yes	Yes
5	Yes	Yes	Yes	No - Gender, age, and medical insurance types were used as	Yes

Study No.	Q1	Q2	Q3	Q4	Q5
				subgroups for the analysis. They were not controlled to explore seasonal effects. Other possible confounders (e.g., comorbidities) were not collected.	
6	Yes	Yes	Can't tell - There was no information about completeness of outcome data.	Yes	Yes
7	Yes	Yes	Yes	Yes	Yes
8	Yes	Yes	Can't tell - There was no information about completeness of each outcome measurement. In many parts, outcomes were reported in percentage without absolute numbers.	Yes	Yes
9	Yes	Yes	Can't tell - There was no information about completeness of each outcome measurement. It was observed that the number of observations changed for different measures.	Yes	Yes
10	Yes	Yes	Can't tell - There was no information about completeness of outcome data.	Yes	Yes

Study No.	Q1	Q2	Q3	Q4	Q5
11	Yes	Yes	Can't tell - Not enough information to tell. The authors only noted that "After disregarding samples with missing information, 83,228 observations are used in the empirical analysis." The number of missing data was not known.	Yes	Yes
12	Yes	Yes	Yes	Yes	Yes
13	No - For Japan, the authors used the Diagnosis Procedure Combination (DPC) database which was a nationwide inpatient database of approximately 1000 participating DPC hospitals and covers approximately 50% of all acute-care admission in Japan. There was no information about whether non-DPC participating hospitals were different from the participated ones.	Yes	Yes	Yes	Yes
14	Yes	Yes	Yes	Yes	Yes

Remark: We agree on an acceptable complete outcome at 90%

Summary of first-round quality rating by two researchers (number of studies)

Questions	1	2	3	4	5
**WW**					
**Yes**	11	13	4	13	14
**No**	3	0	0	1	0
**Can't tell**	0	1	10	0	0
**SV**					
**Yes**	14	11	7	13	13
**No**	0	0	2	1	1
**Can't tell**	0	3	5	0	0

First-round Quality Rating Report Sheet

Study No.	Questions	1	2	3	4	5
**WW**						
**1**	**Yes**	1			1	1
	**No**					
	**Can't tell**		1	1		
	**Comments**		The variables and selected models were appropriate for answering the research question. The outcomes were measured with a reliability test. However, the limitation of this paper mentioned that this study is subject to measurement error due to the survey data based on respondents' self-reported information; no information is available to assess the validity of the data.	No information		
**2**	**Yes**	1	1	1	1	1
	**No**					
	**Can't tell**					
	**Comments**			92.6% completed data (9329/10078)		

Study No.	Questions	1	2	3	4	5
**3**	**Yes**	1	1	0	1	1
	**No**					
	**Can't tell**			1		
	**Comments**			No information		
**4**	**Yes**	1	1	1	1	1
	**No**					
	**Can't tell**					
	**Comments**			97.4% completed data (7836/8049)		
**5**	**Yes**	1	1		0	1
	**No**				1	
	**Can't tell**			1		
	**Comments**		Most parts are good except for those related to Spring Festival, as the authors noted "Since the date of the Spring Festival is not fixed like the National Day holiday period, the statutory holiday intervals of the Spring Festival 2017 was 2.18–2.24 (equivalent to the eighth week) in 2015, 2.7–2.13 (equivalent to the sixth week) in 2016, and 1.27–2.2	No information	Gender, age, and medical insurance types were used as subgroups for the analysis. They were not controlled to explore seasonal effects. Other possible confounders were not collected.	

Study No.	Questions	1	2	3	4	5
			(equivalent to the fifth week) in 2017. When we calculated the seasonal index on a weekly basis, there were inevitable errors due to the shifting Spring Festival dates in the calculation of the seasonal index of the Spring Festival holiday week, causing an overestimate of the seasonal index for the Spring Festival holiday week. In fact, during the Spring Festival holiday, the number of outpatient visits decreased sharply, and the actual holiday effect was larger than the value we presented."			
**6**	**Yes**	1	1		1	1
	**No**					
	**Can't tell**			1		
	**Comments**			No information		
**7**	**Yes**	1	1		1	1
	**No**					
	**Can't tell**			1		

Study No.	Questions	1	2	3	4	5
	**Comments**			No information		
**8**	**Yes**		1		1	1
	**No**	1				
	**Can't tell**			1		
	**Comments**	262/497 districts respond to the questionnaire. There were differences in some characteristics and utilisation profiles between the responded and non-responded districts although the authors noted that "Despite some of these differences, we find no evidence that sample selection bias affects our estimation results."		No information		
**9**	**Yes**		1		1	1
	**No**	1				
	**Can't tell**			1		
	**Comments**	1. Out of a total of 442 districts that were contacted, 262 districts responded (60 percent).<br>2. The two IDHS surveys sampled		No information		

Study No.	Questions	1	2	3	4	5
		children from 234 of the 262 districts that responded to the DHO Survey				
**10**	**Yes**	1	1		1	1
	**No**					
	**Can't tell**			1		
	**Comments**			No information		
**11**	**Yes**	1	1		1	1
	**No**					
	**Can't tell**			1		
	**Comments**			Not enough information on how much data was missing. The authors only noted that "After disregarding samples with missing infor-mation, 83,228 observations are used in the empirical analysis."		
**12**	**Yes**	1	1		1	1
	**No**					
	**Can't tell**			1		
	**Comments**			No information		
**13**	**Yes**		1	1	1	1

Study No.	Questions	1	2	3	4	5
	**No**	1				
	**Can't tell**					
	**Comments**	For Japan, the authors used the Diagnosis Procedure Combination (DPC) database which was a nationwide inpatient database of approximately 1000 participating DPC hospitals and covers approximately 50% of all acute-care admission in Japan. There was no information about whether non-DPC participating hospitals were different from the participated ones.				
**14**	**Yes**	1	1	1	1	1
	**No**					
	**Can't tell**					
	**Comments**					
**SV**						
**1**	**Yes**	1		1	1	1
	**No**					
	**Can't tell**		1			

Study No.	Questions	1	2	3	4	5
	Comments		The variables and selected models were appropriate for answering the research question. The outcomes were measured with a reliability test. However, the limitation of this paper mentioned that this study is subject to measurement error due to the survey data based on respondents' self-reported information; no information is available to assess the validity of the data.			
2	Yes	1	1	1	1	1
	No					
	Can't tell					
	Comments	The limitation of the sample was mentioned in last part of the paper.				
3	Yes	1	1	1	1	1
	No					
	Can't tell					
	Comments			Uninsured people were excluded.		

Study No.	Questions	1	2	3	4	5
**4**	**Yes**	1			1	1
	**No**			1		
	**Can't tell**		1			
	**Comments**		The variables and selected models were appropriate for answering the research question. However, there was no validity and reliability tests for interested outcomes.	There were some missing data.		
**5**	**Yes**	1	1	1	1	1
	**No**					
	**Can't tell**					
	**Comments**			Using patient database, so, no need the information of non-response bias. Also, the number of population in the methodology section was similar to the information presented in the result.		
**6**	**Yes**	1		1	1	1
	**No**					
	**Can't tell**		1			
	**Comments**		The variables and selected models were			

Study No.	Questions	1	2	3	4	5
			appropriate for answering the research question. The outcomes were measured with reliability tests. However, there were some omitted variables mentioned in the limitation of the study.			
7	Yes	1	1	1	1	1
	No					
	Can't tell					
	Comments		Utilisation in this study was the patients choose to use health services in the different types of healthcare providers (not utilisation rate)	Using patient database, so, no need the information of non-response bias. Also, the number of samples in the methodology section was similar to the information presented in the result.		
8	Yes	1	1		1	1
	No					
	Can't tell			1		
	Comments			Some districts did not provide the data. However, the authors mentioned that results		

Study No.	Questions	1	2	3	4	5
				were not affected by sample selection bias.		
9	Yes	1	1		1	1
	No					
	Can't tell			1		
	Comments			High non-response rate, but the authors defended that it was no evidence of sample selection bias affecting our estimation results		
10	Yes	1	1	1	1	1
	No					
	Can't tell					
	Comments					
11	Yes	1	1		1	1
	No					
	Can't tell			1		
	Comments			There were some missing data.		
12	Yes	1	1		1	1
	No			1		
	Can't tell					
	Comments			The number of respondents was less than the sample of		

Study No.	Questions	1	2	3	4	5
				women aged 30-59 years.		
**13**	**Yes**	1	1			
	**No**				1	1
	**Can't tell**			1		
	**Comments**			No information of completed data (in total)		
**14**	**Yes**	1	1		1	1
	**No**					
	**Can't tell**			1		
	**Comments**			No information of completed data (in total). The limitation of the study indicated that the data furnished to the OECD was limited.		
